# 3D hydrogel environment rejuvenates aged pericytes for skeletal muscle tissue engineering

**DOI:** 10.3389/fphys.2014.00203

**Published:** 2014-05-30

**Authors:** Claudia Fuoco, Elena Sangalli, Rosa Vono, Stefano Testa, Benedetto Sacchetti, Michael V. G. Latronico, Sergio Bernardini, Paolo Madeddu, Gianni Cesareni, Dror Seliktar, Roberto Rizzi, Claudia Bearzi, Stefano M. Cannata, Gaia Spinetti, Cesare Gargioli

**Affiliations:** ^1^Department of Biology, University of Rome Tor Vergata Rome, Italy; ^2^IRCCS MultiMedica Milan, Italy; ^3^Stem Cell Lab, Department of Molecular Medicine, Sapienza University of Rome Rome, Italy; ^4^Humanitas Clinical Research Center Milan, Italy; ^5^Experimental Cardiovascular Medicine, University of Bristol Bristol, UK; ^6^IRCCS Fondazione Santa Lucia Rome, Italy; ^7^Faculty of Biomedical Engineering, Technion-Israel Institute of Technology Haifa, Israel; ^8^Cell Biology and Neurobiology Institute, National Research Council of Italy (CNR) Rome, Italy

**Keywords:** stem cells, perycite, skeletal muscle, myogenic differentiation, tissue engineering, PEG-firbinogen, biomaterials

## Abstract

Skeletal muscle tissue engineering is a promising approach for the treatment of muscular disorders. However, the complex organization of muscle, combined with the difficulty in finding an appropriate source of regenerative cells and in providing an adequate blood supply to the engineered tissue, makes this a hard task to face. In the present work, we describe an innovative approach to rejuvenate adult skeletal muscle-derived pericytes (MP) based on the use of a PEG-based hydrogel scaffold. MP were isolated from young (piglet) and adult (boar) pigs to assess whether aging affects tissue regeneration efficiency. *In vitro*, MP from boars had similar morphology and colony forming capacity to piglet MP, but an impaired ability to form myotubes and capillary-like structures. However, the use of a PEG-based hydrogel to support adult MP significantly improved their myogenic differentiation and angiogenic potentials *in vitro* and *in vivo*. Thus, PEG-based hydrogel scaffolds may provide a progenitor cell “niche” that promotes skeletal muscle regeneration and blood vessel growth, and together with pericytes may be developed for use in regenerative applications.

## Introduction

Adult skeletal muscle tissue has a remarkable capability to regenerate after injury, although this property is related to damage entity. Muscle harm evokes the activation of different mononucleated cell populations that, in response to myofiber degeneration, proliferate and differentiate into myocytes, enabling muscle regeneration (Carlson, 1973). The main actors of this process are muscle satellite cells, quiescent resident stem cells located between the sarcolemma and the basal lamina. This specific microenvironment, often referred to as a “cellular niche,” is instrumental in keeping the satellite cell in a quiescent state, but promotes activation of the muscle progenitor cells after injury. In addition, many other cell types, either residing in different muscle compartments or attracted from the circulation following injury, contribute to the regeneration process (Bentzinger et al., 2013).

Similarly to the niches of specialized cells that have been described in skeletal muscle and bone marrow, the wall of skeletal muscle vessels is believed to possess a niche of regenerative mesenchymal cells, namely pericytes (Birbrair et al., 2013). Pericytes play a crucial role in the control of angiogenesis, both in the early stages and in the stabilization of the nascent structure. This effect is exerted via direct and paracrine factor-mediated interaction with the endothelium. In particular, crucial biological functions of endothelial cells (EC) such as migration, proliferation, permeability, and contractility, are affected by pericytes (Armulik et al., 2005; Ribatti and Crivellato, 2011; Dulmovits and Herman, 2012). Pericytes reside in anatomical proximity to ECs, sharing the basement membrane, making specialized junctions, and surrounding them at the tip of capillaries, ready to guide the vasculogenic process. The EC:pericyte ratio varies throughout the body, ranging from 1:1 in neural tissues to 10:1 in skeletal muscles (Armulik et al., 2011). In response to pro-angiogenic conditions, pericytes contribute to the mobilization of ECs, promoting the formation and stabilization of the nascent vessel until the accomplishment of the process preventing microvascular regression. Their ability to interact with ECs and to regulate angiogenesis as well as their documented capacity of multilineage mesenchymal differentiation render pericytes suitable for investigations in the field of tissue regeneration and promotion of angiogenesis in ischemic diseases and in wound healing (Abou-Khalil et al., 2010). In this respect, pericytes resident in the adventitia of vessels, such as the saphenous vein, have been shown by our group to represent a potent class of pro-angiogenic cells *in vitro* and *in vivo* in experimental models of ischemia (Campagnolo et al., 2010; Katare et al., 2011).

We address here the potential of pericytes resident in adult skeletal muscle to support muscle differentiation and angiogenesis *in vitro* and *in vivo*. The underlying hypothesis is that the bi-directional differentiation potential of skeletal muscle-derived pericytes (MP)—i.e., for vasculogenesis and myogenesis—may represent a plus in the development of new regenerative approaches for ischemic or wounded tissues. Although their contribution to the *in vivo* muscle repair process seems to be marginal when compared with that of satellite cells, their noteworthy myogenic capability makes them attractive as alternative source of myogenic progenitors for stem cell-mediated muscle regeneration approaches (Sirabella et al., 2013). Indeed, skeletal muscle tissue is unable to duly repair in response to a severe injury, so reconstructive strategies, such as autologous muscle transplantation or intra-muscular injection of progenitor cells, are needed to recover the damaged area. Moreover, ageing negatively affects muscle regeneration and myogenic stem cell potential, resulting in muscular tissue pauperization or sarcopenia (Carosio et al., 2011; García-Prat et al., 2013). In particular, several studies have demonstrated that aged muscle satellite cells present functional alterations, such as a decreased capacity to form colonies, when cultured *in vitro*, and an impaired capability to activate and proliferate in response to damage (Conboy et al., 2003; Baj et al., 2005; Shefer et al., 2006; Day et al., 2010; Shadrach and Wagers, 2011). Aging also significantly affects the capacity of muscle satellite cells to differentiate, to fuse into myotubes and to replenish reserve pools in culture (Carlson and Faulkner, 1996; Grounds, 1998; Shavlakadze et al., 2010).

Tissue engineering is a novel discipline aiming to mimic organogenesis. In principle, one could consider generating new skeletal muscle tissue *in vitro* and implanting it *in vivo* to replace severely injured muscle. The skeletal muscle engineering approach contemplates two main components: stem cells and a biomaterial, ensuring a regenerative capacity and tissue scaffolding, respectively. In order to guarantee a suitable *in vivo* support for new tissue generation, the biomaterial must have specific characteristics: it must be inert, with minimal cytotoxicity, resorbable by cell-mediated biodegradation processes and modifiable, its stiffness included (Rossi et al., 2010; Fuoco et al., 2012; Rizzi et al., 2012; Seliktar, 2012). Extracellular matrix (ECM) constituents have been used as a model and as components for scaffold materials, guaranteeing skeletal muscle microenvironment reconstitution and muscular regeneration amelioration. Several natural or synthetic polymers have also been studied as biomaterials in rats and dogs for striated muscle regeneration, mainly supporting cardiac repair; nevertheless, just a few have been tested in human clinical experimentation (Zammaretti and Jaconi, 2004; Habib et al., 2011).

Here, we report on the effects of polyethylene glycol (PEG)–fibrinogen (PF) on the skeletal muscle regeneration capacity of pericytes. We found that seeding onto a PF scaffold recuperated the reduced myogenic/vasculogenic ability of aged pericytes *in vitro*, allowing these cells to generate a vascularized muscle *in vivo* that was indistinguishable from that formed with the use of younger cells.

## Methods

### Muscle pericyte isolation

Muscle biopsies from piglet and boar (Large White X Landrace) were kindly provided by Dr. La Torre of the animal house facility of Tecnopolo Castel Romano, Italy. Skeletal muscle biopsies (approximately 2 cm^3^) were sterilely isolated from porcine quadriceps, soaked in PBS and processed within 24 h. Muscular tissue was finely minced with tweezers until no intact muscle pieces could be distinguished. The minced tissue was digested for 45 min with collagenase II (0.1 mg/ml, GIBCO) at 37°C. After filtration through a 70 μm strainer, cells were washed in PBS and plated in DMEM GlutaMAX (GIBCO) supplemented with heat-inactivated 20% fetal bovine serum (FBS), 100 iu/ml penicillin and 100 mg/ml streptomycin on plastic dishes. We selected pericytes by their capacity to grow in this relatively poor condition at low cell confluence (0.1–1 × 10^4^ cell/cm^2^), as opposed to mesoangioblasts (Tonlorenzi et al., 2007). The first fibroblast colony-forming units (CFU-F) formed 7–10 days after seeding (Sacchetti et al., 2007), after which pericyte colonies were expanded and tested for their myogenic and angiogenic potential. For myogenic differentiation analysis, swine muscle-derived pericytes (MP) were cultured on plastic dishes as above cited for 24–48 h, until 80% confluent, then medium was replaced with DMEM supplemented with 5% horse serum (differentiation medium) to promote muscle differentiation.

### Immunofluorescence

Cells and tissue were fixed in 2% PFA and processed for histology and immunocytochemistry as previously described (Gargioli et al., 2008). Briefly, MP were cultured in fibronectin-coated 8-well chamber slides (Nunc) as described above. After fixation with 2% PFA for 10 min, cells were incubated overnight at 4°C with the following primary antibodies: anti-α-smooth muscle actin (SMA; Dako), platelet-derived growth factor receptor beta (PDGFR-β; Cell Signaling Technology), chondroitin sulfate proteoglycan (NG2; Merk Millipore) and myosin heavy chain (MyHC; DHSB), diluted in accordance with the manufacturers' instructions. Cells were then incubated with the appropriate secondary fluorophore-conjugated antibody. Nuclei were stained with DAPI. Fluorescence photomicrographs were taken with an Axio Observer A1 microscope equipped with a fluorescence detection system at 20 × magnification. Imaging analysis was performed with AxioVision Imaging System software (Zeiss).

### Immunoblotting

Tissue samples were pulverized in liquid nitrogen and then immediately homogenized in RIPA buffer (20 mM Tris/HCl, pH 7.4, 5 mM EDTA, 0.1% SDS, 1% NP40, 1% NaDOC, and Roche protease inhibitor cocktail). Homogenates were centrifuged at 12,000 g for 10 min at 4°C to discard nuclei and cellular debris. Protein concentration was determined with bicinchoninic acid (BCA) protein assay (Pierce) using bovine serum albumin (BSA) as the standard. Total homogenates were separated by sodium dodecyl sulfate–polyacrylamide gel electrophoresis (SDS–PAGE) with a concentration opportunely chosen on the base of molecular weight of the proteins analyzed. For Western blot analysis, proteins were transferred onto Immobilon membranes (Amersham), saturated with 5% non-fat dry milk (Biorad) in 0.1% Tween-20 (Sigma) PBS (blocking solution) and hybridized with 1:5 MF20 mouse monoclonal antibody against MyHC (DHSB) or with 1:5000 anti-GAPDH (clone 71.1, Sigma) for 1 h at RT. The filters were washed three times (15 min each at RT) with wash solution (0.1% Tween-20 in PBS) and then reacted with anti-mouse, anti-rat, or anti-rabbit secondary antibody conjugated with 1:3000 horseradish peroxidase IgG (Biorad) for 1 h at RT, washed three times and finally visualized with ECL (Amersham). Optical density (OD) was calculated with ImageJ software.

### Tube forming assay

Human umbilical vein endothelial cells (HUVEC) and MP were seeded in 8-well Permanox chamberslides (Nunc) coated with 150 μl Matrigel (3D; BD Biosciences), either alone (3.75 × 10^4^ cells/well) or as a 1:4 MP-to-HUVEC ratio co-culture, in medium supplemented with 0.1% BSA. Cells were incubated for at least 5 h post-seeding to allow tube formation. Images of the networks were taken with a 5× objective (Axio Observer A1, Zeiss, Germany). Images of branches at the nodes of a ramification of formed tubes were taken with a 20× objective. All experiments were conducted in duplicates.

### Creation of PEG-fibrinogen constructs

The PEG-fibrinogen precursor solution was prepared and photo-polymerized as described elsewhere (Fuoco et al., 2012). We prepared PEG hydrogels containing MP by mixing a PBS cell suspension with PEGylated fibrinogen precursor solution containing 0.1% Igracure 2959 photoinitiator (Ciba Specialty Chemicals) in order to have a final concentration of 8 mg/ml. We added 100 μl of the suspension into cylindrical silicon molds and placed them under a long-wave UV lamp (365 nm, 4–5 mW/cm^2^) for 5 min in a laminar flow hood. DMEM culture medium (containing 20% FBS) was added immediately to the polymerized hydrogels to ensure cell growth. The plugs were cultured for 24 h in serum-supplemented growth medium and then transferred into serum-depleted differentiation medium for 5 days in order to promote muscle fiber formation. For *in vivo* experiments, molds were immediately implanted subcutaneously in the backs of mice.

### Implantation of constructs

Two-month-old male RAG2/γChain immunocompromised mice were anesthetized with an intramuscular injection of physiologic saline (10 ml/kg) containing ketamine (5 mg/ml) and xylazine (1 mg/ml) and then PF constructs containing 1.5 × 10^6^ swine-derived MP implanted subcutaneously in their backs (one side received a construct seeded with piglet MP, and the other side a construct seeded with boar MP). In order to ensure good placement of the constructs, we performed a limited incision on the medial side of the back, separated the dorsal muscle from the skin, placed the plug as desired and then sutured the wound. Mice were sacrificed 30 days after implantation for molecular and morphological analysis. Experiments on mice were conducted according to the rules of good animal experimentation I.A.C.U.C. n° 432, March 12 2006.

### Statistical analysis

Continuous variables are expressed as means ± standard error (SEM). Statistical significance was tested using GraphPad Prism 5 software and applying Student's *t*-test or One-Way ANOVA followed by Bonferroni multiple comparison post-test or Kruskal–Wallis test if data had a skewed distribution.

## Results

### Aging does not impact on morphology, CFU capacity or marker expression of swine muscle-derived pericytes

**Figure 1:**
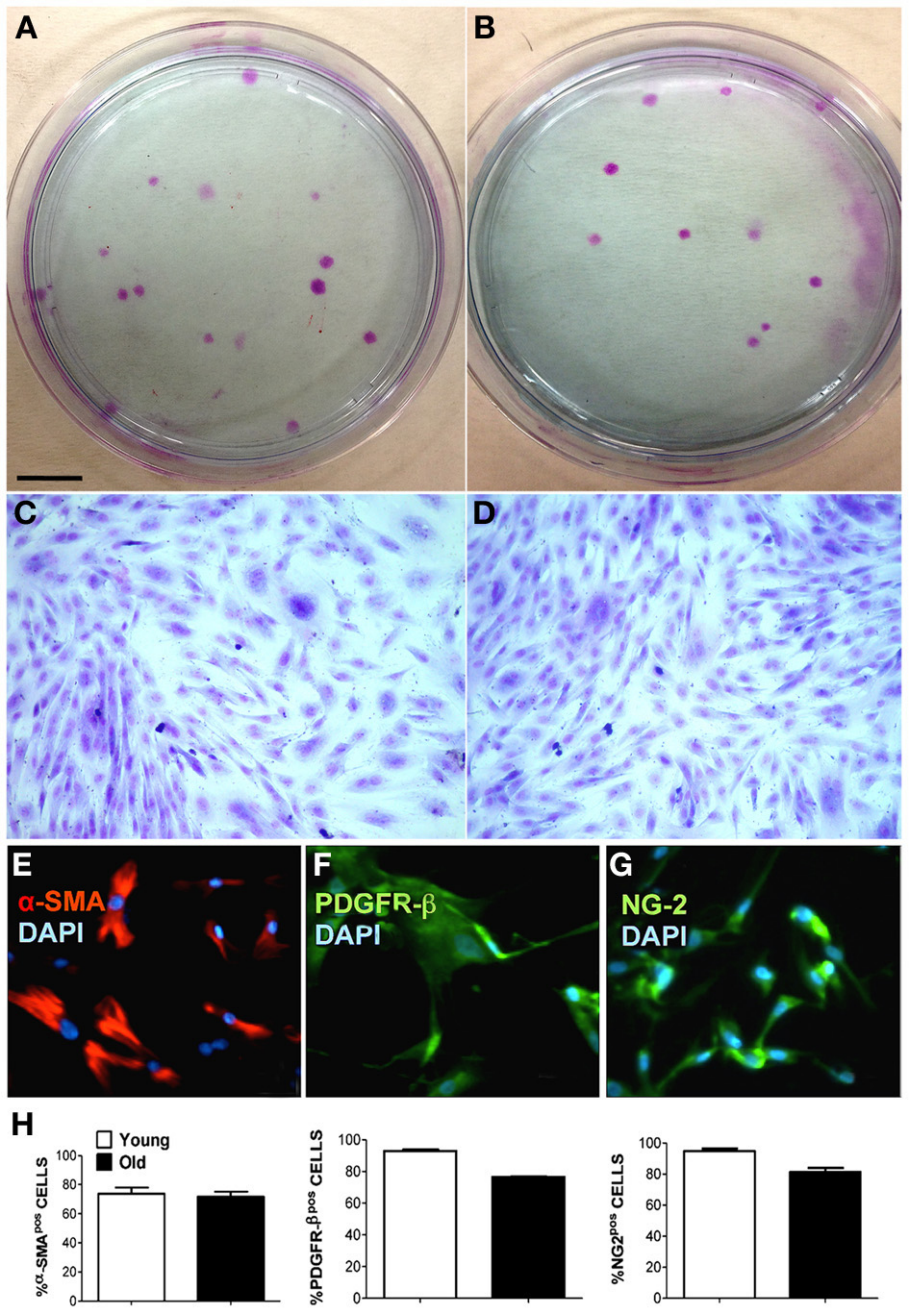
**Characterization of muscle-derived pericytes (MP) from swine**. MP were analyzed for their behavior and their expression of characteristic pericyte markers to define their cellular identity. **(A,B)** Young **(A)** and old **(B)** swine-derived MP showing fibroblast colony-forming units (CFU-F) when seeded at low confluence. Giemsa staining on plastic 9 cm Petri dishes. **(C,D)** Single CFU-F morphology of young **(C)** and adult **(D)** MP, presenting identical spindle-like morphology. **(E–G**) Representative immunofluorescence staining of young MP showing positivity (pos) for alpha-smooth muscle actin (α-SMA) **(E)**, chondroitin sulfate proteoglycan (NG-2) **(G)**, and platelet-derived growth factor receptor beta (PDGFR-β) **(F)**. Nuclei were counterstained with 4, 6-diamidino-2-phenylindole (DAPI). **(H)** Average data scored from immunofluorescence in **(E–G)**. Scale bar: **(A,B)** 1 cm; **(C–G)** 15 μm.

MP were isolated and cultured as described in the Methods section. Freshly isolated MP were cultured in plastic Petri dishes at a low density (0.1–1 × 10^4^ cell/cm^2^) in order to evaluate CFU-F capacity and for immunofluorescence staining using specific pericyte antigenic markers. We found that piglet- and boar-derived MP had a similar CFU-F capacity and cell shape (Figures 1A–D). Isolated swine MP were positive for typical pericyte markers, such as SMA, PDGFR-β and NG2 (Figures 1E–G), and there was no difference in the percentage of positive cells from piglets and boars (Figure 1H). These findings suggested that age does not affect MP morphology or CFU-F capability.

### Adult muscle-derived pericytes have impaired angiogenic and myogenic differentiation capacities *in vitro*

**Figure 2:**
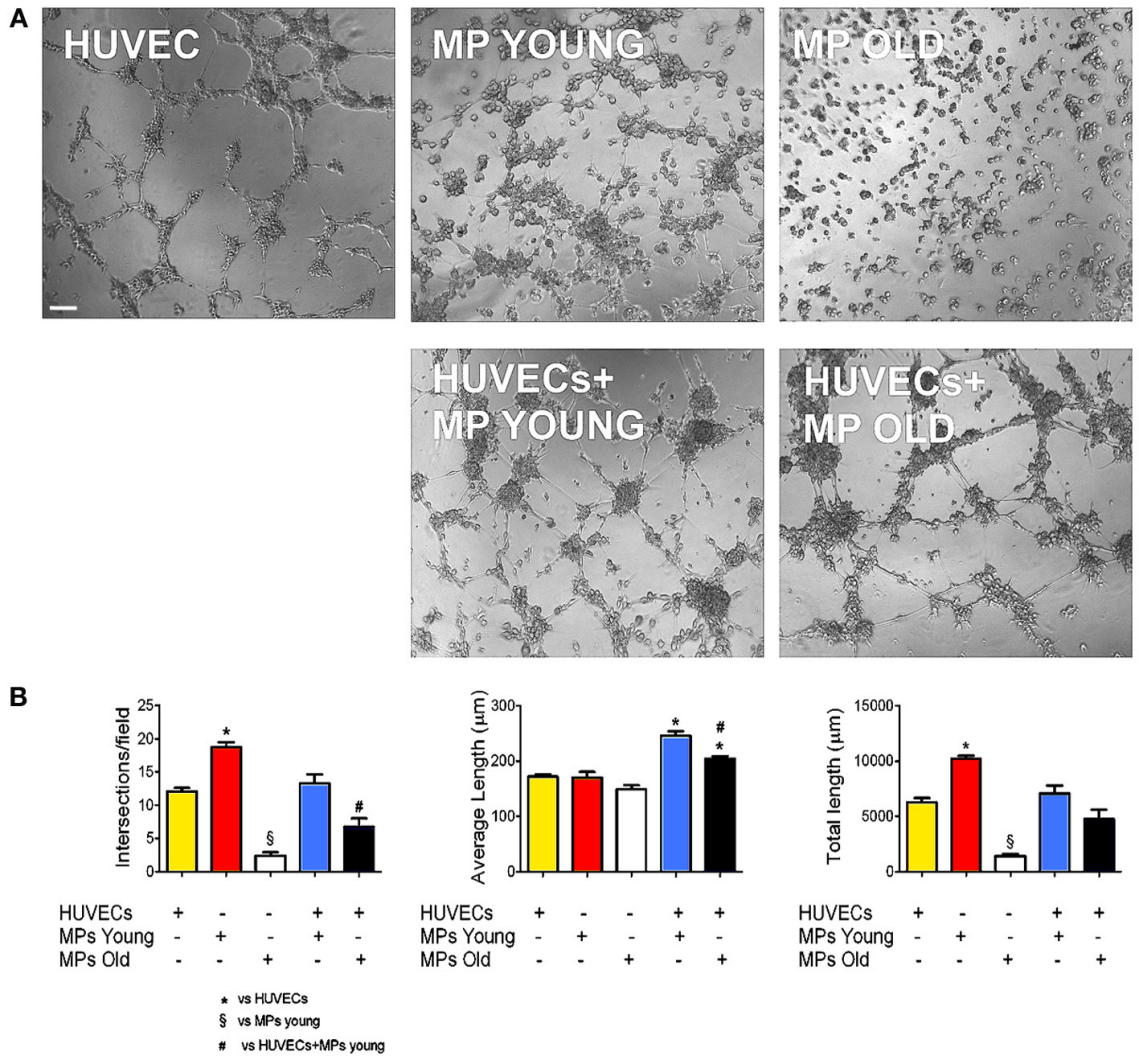
**Ability of MP to form capillary-like structures *in vitro***. Endothelial cell networking *in vitro* on Matrigel. **(A)** Phase-contrast microphotographs showing human umbilical vascular endothelial cell (HUVEC) and muscle-derived pericyte (MP) capacity to form networks on Matrigel, alone and in co-culture (1:4 MP:HUVEC ratio). **(B)** The efficiency of tube formation was quantified as number of intersections, average, and total tube length. Values are expressed as mean ±s.e.m. of *n* = 3 donor/group assayed in duplicate. ^*^p < 0.05 vs. HUVECs alone; ^§^*p* < 0.05 vs. young MP; ^#^*p* < 0.05 vs. HUVECs + young MP. Scale bar: 100 μm.

**Figure 3:**
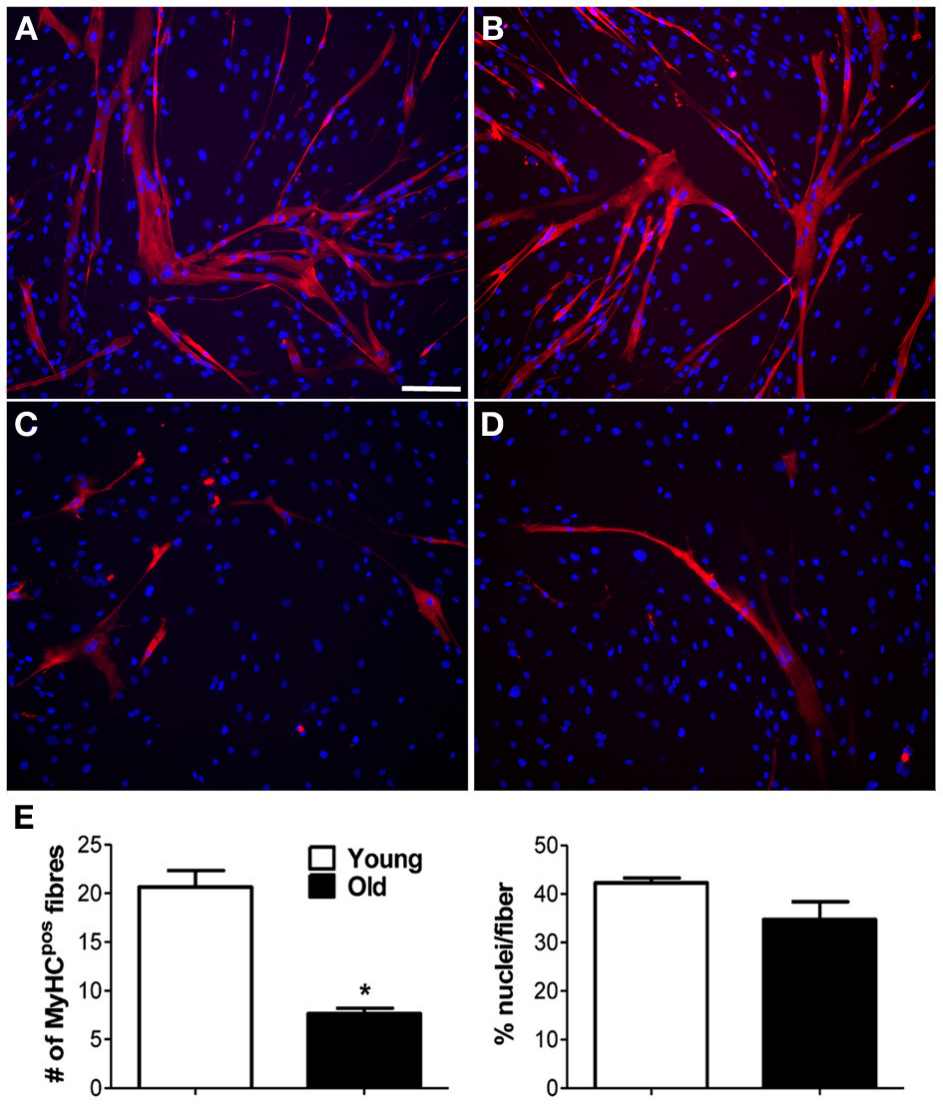
**Myogenic capability of MP *in vitro***. Myogenic ability was assessed for muscle-derived pericytes (MP). **(A–D)** Immunofluorescence staining with anti-myosin heavy chain (MyHC) antibody. Representative images showing MyHC-positive (pos) myotubes (red) formed from young **(A,B)** and old **(C,D)** MP, demonstrating that age affects MP muscle differentiation capacity. Nuclei were counterstained with 4,6-diamidino-2-phenylindole (DAPI). **(E)** Average data extracted from **(A–D)** immunolabeling. The number of positive fibers was quantified with respect to the total number of nuclei. The efficiency of cell fusion into syncytial myofibers was measured as percentage of nuclei per fiber. The analysis was done on five pictures from three donor/group. Values are expressed as mean ±s.e.m. ^*^p < 0.05 vs. young MP. Scale bar: **(A–D)** 50 μm.

Adequate blood supply is indispensable to guarantee cell survival and engraftment in skeletal muscle tissue engineering applications. Because pericytes are active players in capillary sprouting and vessel stabilization, we assessed the *in vitro* capacity of MP to form networking structures and to cooperate with endothelial cells (HUVEC) to form capillary-like structures (Figure 2A). We found that piglet MP had an enhanced ability to form capillary-like structures on Matrigel compared with HUVEC (Figure 2B). In boar MP, this ability was severely hampered and was not recuperated by co-culture with HUVEC. The estimates of the average number of intersections and length of capillary-like structures consistently pointed to a negative impact of age on the ability of porcine MP to support vessel formation. MP can differentiate into myoblasts that fuse into multinucleated fibers (Cappellari and Cossu, 2013). Together with the formation of capillary like-structures, this property is crucial for the reparative function of MP. We therefore tested whether aging had an effect on this ability too. High confluency and serum depletion were used to promote myogenic differentiation in MP (Péault et al., 2007). Piglet-derived MP differentiated spontaneously into multinucleated myotubes when they became confluent (Figures 3A,B), an ability that was markedly greater than in adult MP (Figures 3C,D). In fact, we found that the number of MyHC-positive fibers formed by boar MP was significantly lower (Figure 3E). However, this was not accompanied by a decreased fusion index (i.e., nuclei/fiber), suggesting that a lower percentage of adult MP retained an efficient myogenic ability.

### PEG-fibrinogen improves the myogenic capability of adult MP

**Figure 4:**
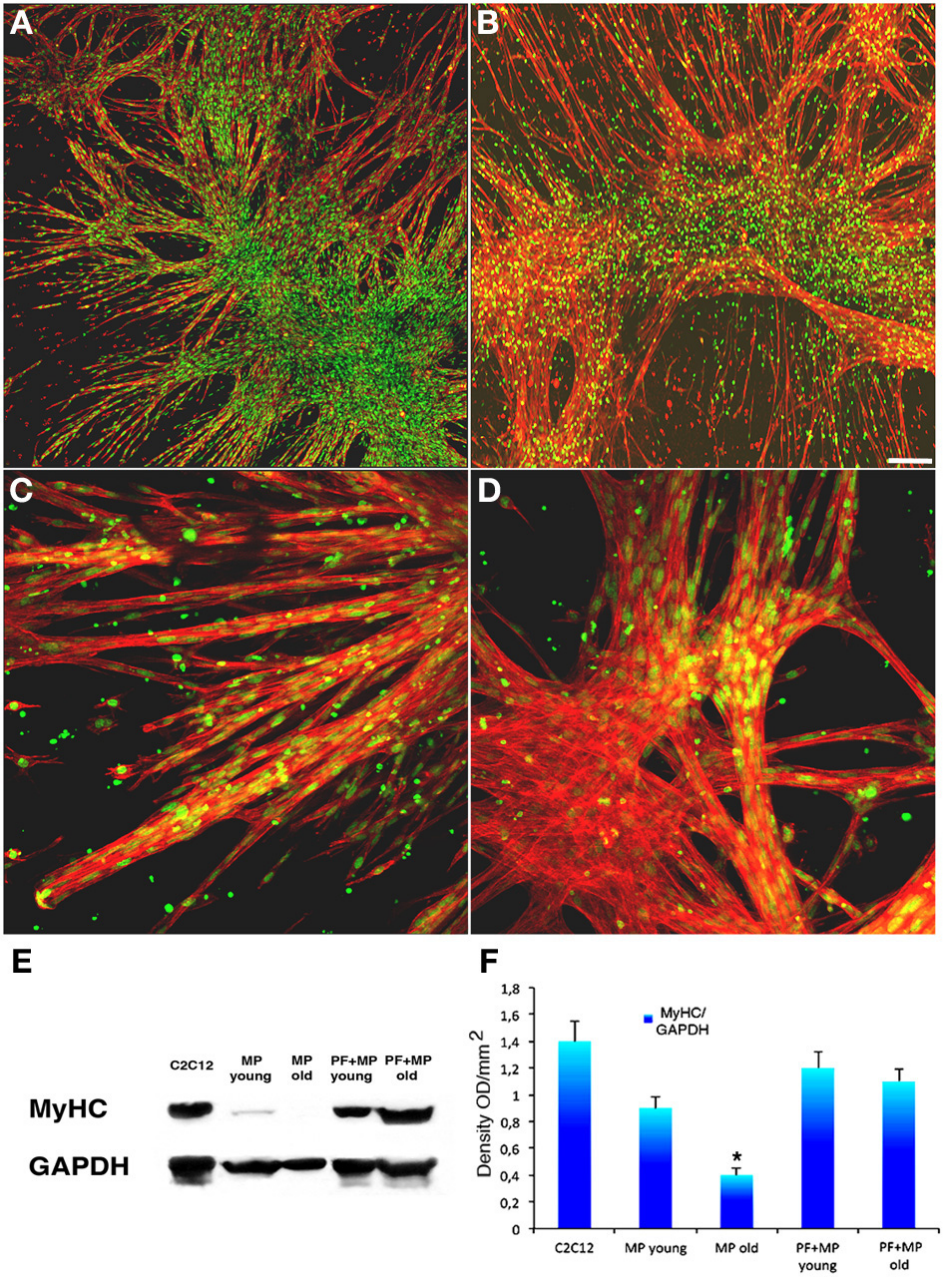
**PEG-fibrinogen re-establishes the myogenic capability of adult MP. (A,B)** Confocal images of immunofluorescence labeling with an antibody against MyHC (red) and SYTOX Green for nuclei (green) on myotubes developed from young **(A)** and adult **(B)** pericytes cultured on a PEG-fibrinogen scaffold (5 days). **(C,D)** Enlarged view respectively of young **(C)** and adult **(D)** pericytes cultured on a PEG-fibrinogen showing multinucleated muscle fibers. **(E)** Representative Western blot of protein extracts from differentiated C2C12 cells as control and young and adult muscle-derived pericytes (MP) cultured on plastic or encapsulated in a PEG-fibrinogen scaffold (PF+), showing myosin heavy chain (MyHC) expression. **(F)** Densitometric analysis of three different Western blots quantifying the level of expression of MyHC. mean ±s.e.m., normalized to GADPH. ^*^p < 0.05 vs. Young MP. Scale bar: **(A,B)** 50 μm; **(C,D)** 10 μm.

**Figure 5:**
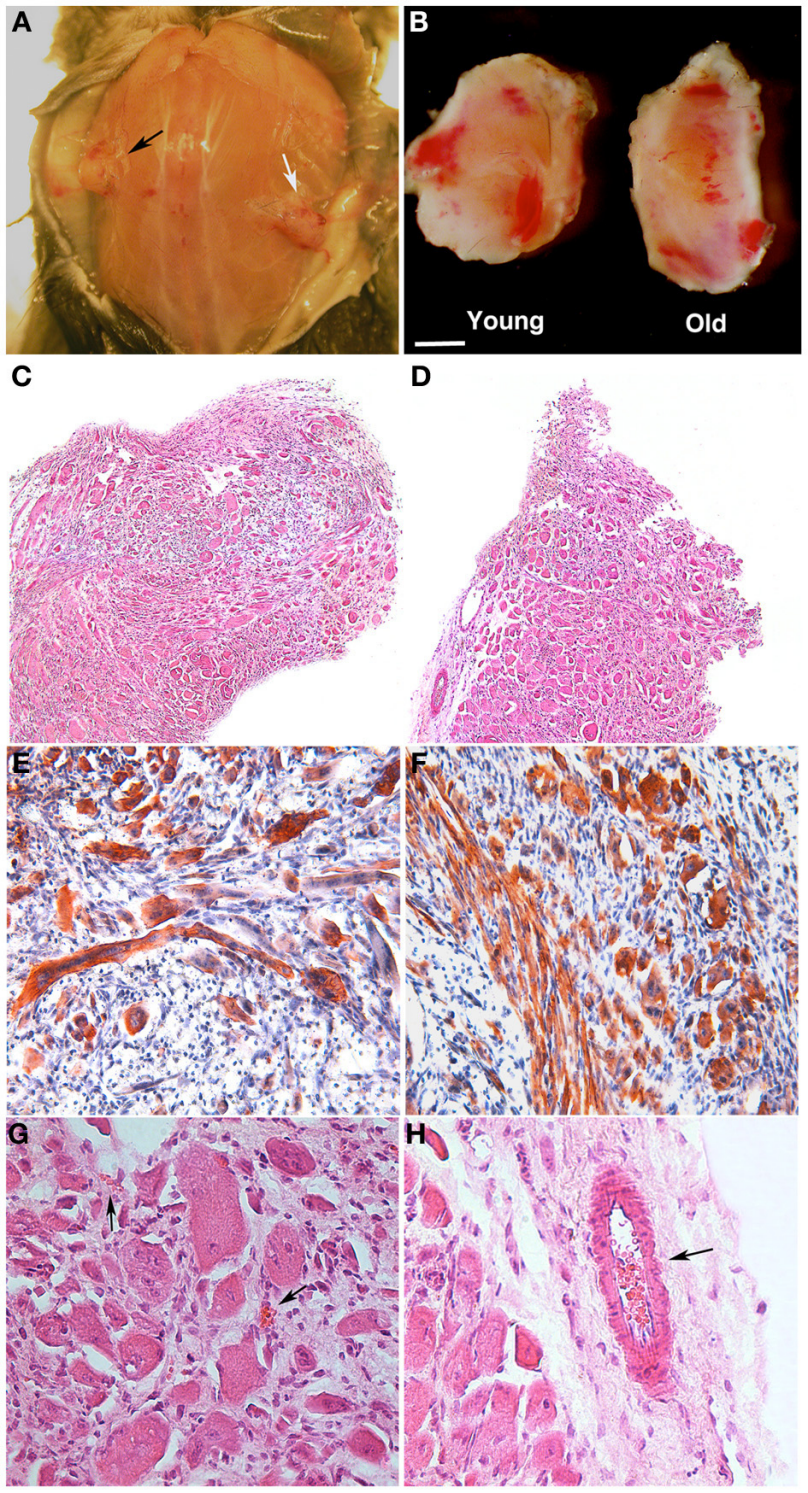
**PEG-fibrinogen-pericyte constructs form ectopic muscle *in vivo***. Muscle-derived pericytes (MP) seeded onto PEG-fibrinogen scaffolds (MP + PF) were implanted subcutaneously in immunocompromised mice and extracted 30 days after grafting (*n* = 6). **(A,B)** Macroscopic comparison of subcutaneous MP + PF implants loaded with 5 × 10^5^ young MP (black arrow) and 5 × 10^5^ adult MP (white arrow) **(A)**, revealing an ectopic, vascularized tissue-like formation **(B)**. **(C,D)** Hematoxylin/eosin stained sections of ectopic tissue revealing similar muscular-like structures generated from young **(C)** and adult **(D)** MP. **(E,F)** Immunohistochemistry on young- **(E)** and old- **(F)** MP-derived ectopic tissue showing muscle fiber positive for myosin heavy chain (red/brown). Nuclei stained with hematoxylin (blue). **(G,H)** Enlarged view of, respectively **(C)** and **(D)**, revealing the presence of vessels (arrows) in a muscle-like tissue. Scale bar: **(A)** 10 mm; **(B)** 2.5 mm; **(C,D)** 300 μm; **(E,F)** 100 μm; **(G,H)** 50 μm.

PF is a relatively novel material that transitions into a gel under a UV trigger (Almany and Seliktar, 2005). It was recently demonstrated that PF has a remarkable influence on myogenic differentiation of progenitor muscle cells by providing a three-dimensional microenvironment suitable for myofiber development (Fuoco et al., 2012; Rizzi et al., 2012). We therefore assessed whether PF could improve the poor myogenic capability of adult MP. To this end, cylindrically shaped silicon molds were filled with 100 μl PF seeded with 5 × 10^5^ MP from piglets or boars, and then exposed to a UV source, as described in the Methods section. The scaffolded MP were cultured for 24 h in serum-supplemented growth medium and then transferred into serum-depleted differentiation medium for 5 days to promote muscle differentiation and myotube formation. Immunofluorescence for MyHC indicated that piglet-derived MP encapsulated in PF had an enhanced myogenic activity, whereas adult MP recovered their myogenic capability almost completely, becoming comparable to that of piglet MP (Figures 4A–D). Western blotting analysis supported the immunofluorescence findings (Figures 4E,F). To assess whether porcine MP could be used as a source of myogenic precursor cells for skeletal muscle tissue engineering, MP–PF constructs were implanted subcutaneously into the backs of immunocompromised mice immediately after the polymerization step, and explanted 30 days later. We found that the PF cylinders seeded with either young or adult MP formed ectopic tissue (Figure 5A) that contained vessel-like structures (Figure 5B). Histological analysis revealed the presence of a tissue with a muscle-like organization (Figures 5C–H) containing MyHC-positive fibers (Figures 5E,F) and blood vessels, probably generated by the recruitment of host EC and/or by host vessels branching into the ectopic tissue (Figures 5G,H).

## Discussion

Over the past few years, we have witnessed a remarkable progress in our understanding of ageing on tissue deterioration and stem cell functionality (Carosio et al., 2011; García-Prat et al., 2013). It is now evident that the stem cell microenvironment—the cellular niche—plays a fundamental role in muscle progenitor cell rejuvenation, as elegantly demonstrated by hetero-parabiosis and hetero-grafting skeletal muscle experiments (Conboy et al., 2005). Thus, regenerative medicine approaches based on the use of adult stem cells could benefit from methods that rejuvenate the cells used. On this point, the present study reports on an innovative strategy to rejuvenate adult muscle progenitor cells. We studied pericytes because they offer a noteworthy advantage over other cell types on account of their bi-directional—vasculogenic and myogenic—commitment capacity, which can be exploited for skeletal muscle tissue engineering. With respect to pericytes from piglets, we found that aged pericytes had an impaired functionality in regards to their myogenic differentiation capacity and ability to support vessel formation. This reduced potential was recuperated by culture on a PEG-fibrinogen scaffold (PF). The PF has been chosen as rejuvenescence inducing matrix on the basis of our foregoing researches revealing the PF to be a very suitable material supporting muscle differentiation (Fuoco et al., 2012). Moreover we strongly believe that the hybrid nature of PF, represented by tunable synthetic PEG and natural fibrinogen molecules as integrant parts of the scaffold, favors cell adhesion, survival and differentiation. Thus PF guarantees a protective and inductive environment promoting swine-derived stem cells rejuvenation by recovering muscle differentiation and angiogenic capabilities of aged porcine perycites. We can speculate that vascularization of ectopic muscle like tissue comes from influence generated by swine derived perycite on host blood vessel recruitment for their intrinsic property to form the suitable “angiogenic niche” and than to retrieve EC for vessel branching and sprouting (Sacchetti et al., 2007; Ribatti et al., 2014). Furthermore recent publications on skeletal muscle tissue engineering demonstrate that exogenous engineered muscle like tissue can be vascularized by host vessel colonization or in case of co-culture with a source of EC (Levenberg et al., 2005; Juhas et al., 2014). These obtained results reflect the effect of a healthy three-dimensional ECM on muscle stem cells. In fact, aging modifies the ECM by increasing endo- and perimysial connective tissue, which alters the mechanical properties of muscle (Seene et al., 2012). Young, healthy ECM has a profound effect on muscle stem cell capabilities compared with an aged, stiffer environment (Antia et al., 2008; Sicari et al., 2012). Therefore, we can speculate that the three-dimensional environment of the PF plugs has a positive action on swine-derived MP by mimicking the stiffness and mechanical cues of young muscle ECM, since evoking into aged MP young-old hetero-parabiosis and /or hetero-grafting promoting swine derived MP rejuvenation. This opens a new scenario for future developments exploiting adult pericytes as myogenic progenitors and an angiogenic trigger to generate engineered muscle for use in humans, overall for the possibility to build with swine derived MP porcine artificial muscle like human size comparable.

## Author contributions

Cesare Gargioli and Gaia Spinetti designed the research and wrote the paper. Claudia Fuoco and Elena Sangalli isolated the cells and carried out most of the experimental work; Roberto Rizzi and Claudia Bearzi helped with data interpretation; Rosa Vono performed cell characterization; Benedetto Sacchetti helped with histological technique and analysis; Dror Seliktar produced and implemented PF for muscle experiments; Sergio Bernardini and Stefano Testa did the histology and immunostaining; Michael V. G. Latronico, Paolo Madeddu, Gianni Cesareni and Stefano Cannata helped with study design, data analysis interpretation, and paper writing.

### Conflict of interest statement

The authors declare that the research was conducted in the absence of any commercial or financial relationships that could be construed as a potential conflict of interest.
