# Corrigendum: 3D hydrogel environment rejuvenates aged pericytes for skeletal muscle tissue engineering

**DOI:** 10.3389/fphys.2019.00199

**Published:** 2019-03-11

**Authors:** Claudia Fuoco, Elena Sangalli, Rosa Vono, Stefano Testa, Benedetto Sacchetti, Michael V. G. Latronico, Sergio Bernardini, Paolo Madeddu, Gianni Cesareni, Dror Seliktar, Roberto Rizzi, Claudia Bearzi, Stefano M. Cannata, Gaia Spinetti, Cesare Gargioli

**Affiliations:** ^1^Department of Biology, University of Rome Tor Vergata, Rome, Italy; ^2^IRCCS MultiMedica, Milan, Italy; ^3^Stem Cell Lab, Department of Molecular Medicine, Sapienza University of Rome, Rome, Italy; ^4^Humanitas Clinical Research Center, Milan, Italy; ^5^Experimental Cardiovascular Medicine, University of Bristol, Bristol, United Kingdom; ^6^IRCCS Fondazione Santa Lucia, Rome, Italy; ^7^Faculty of Biomedical Engineering, Technion-Israel Institute of Technology, Haifa, Israel; ^8^Cell Biology and Neurobiology Institute, National Research Council of Italy (CNR), Rome, Italy

**Keywords:** stem cells, perycite, skeletal muscle, myogenic differentiation, tissue engineering, PEG-firbinogen, biomaterials

In the original article, we neglected to include the funder “European Research Council,” “Grant Number 322749” to GC.

The authors apologize for this error and state that this does not change the scientific conclusions of the article in any way. The original article has been updated.

